# Vertical and temporal variations of soil bacterial and archaeal communities in wheat-soybean rotation agroecosystem

**DOI:** 10.7717/peerj.12868

**Published:** 2022-02-10

**Authors:** Mika Yokota, Yupeng Guan, Yi Fan, Ximei Zhang, Wei Yang

**Affiliations:** 1 Dulwich College Beijing, Beijing, China; 2 Institute of Environment and Sustainable Development in Agriculture, Chinese Academy of Agricultural Sciences, Beijing, China

**Keywords:** Bacteria, Archaea, Vertical distribution, Temporal variations, Soil

## Abstract

Soil microbes are an essential component of terrestrial ecosystems and drive many biogeochemical processes throughout the soil profile. Prior field studies mainly focused on the vertical patterns of soil microbial communities, meaning their temporal dynamics have been largely neglected. In the present study, we investigated the vertical and temporal patterns of soil bacterial and archaeal communities in a wheat-soybean rotation agroecosystem at a depth of millions of sequences per sample. Our results revealed different vertical bacterial and archaeal richness patterns: bacterial richness was lowest in the deep soil layer and peaked in the surface or middle soil layer. In contrast, archaeal richness did not differ among soil layers. PERMANOVA analysis indicated that both bacterial and archaeal community compositions were significantly impacted by soil depth but unaffected by sampling time. Notably, the proportion of rare bacteria gradually decreased along with the soil profile. The rare bacterial community composition was the most important indicator for soil nutrient fertility index, as determined by random forest analysis. The soil prokaryotic co-occurrence networks of the surface and middle soil layers are more connected and harbored fewer negative links than that of the deep soil layer. Overall, our results highlighted soil depth as a more important determinant than temporal variation in shaping the soil prokaryotic community and interspecific interactions and revealed a potential role of rare taxa in soil biogeochemical function.

## Introduction

Soil microbes are an essential component of terrestrial ecosystems and drive many biogeochemical processes throughout the soil profile. They are, therefore, closely linked to soil fertility and crop production (Cao et al., 2012; Fierer, 2017; Jiao et al., 2018; Chaudhary et al., 2021). In agroecosystems, crop roots are mainly distributed in the surface soil layer, though they generally extend below 20 cm, even to depths as deep as 150 cm (Hao et al., 2020). Deep soil is important because it harbors more than two-thirds of the total soil organic carbon and nearly equal amounts of phosphorus (Jobbágy & Jackson, 2000), serving as a key nutrient reservoir for crops. Alternatively, crop roots may absorb water from deep soil during dry seasons when surface soil dries out quickly (Hao et al., 2020). Despite so, previous investigations of soil microbial diversity and community composition are mainly restricted to the surface soil layer (top ∼20 cm), limiting our understanding of microbes’ potential functions in deep soil. Thus, determining the vertical patterns of soil microbial diversity and community composition would improve our understanding of the processes involved in agricultural soil nutrient cycling and contribute to agroecosystem productivity.

The vertical soil profile is highly heterogeneous, with drastic differences in soil texture, nutrient availability, and organic carbon levels among different soil depths (Kim et al., 2016; Jiao et al., 2018). Previous surveys of vertical microbial distributions revealed that soil depth could be a key determinant in shaping microbial diversity and community assembly in alpine meadows, forests, and agroecosystems (Eilers et al., 2012; Hao et al., 2020; Xu et al., 2021). However, existing surveys of vertical microbial distributions have mainly employed one-time sampling, only capturing a specific status of soil microbes when soil microbial communities could be highly dynamic and show temporal patterns (Yang et al., 2019; Yang et al., 2020; Kivlin & Hawkes, 2020). In previous meta-analyses, soil microbial biomass and community composition were temporally variable across the globe (Shade et al., 2013; Wardle, 1998). Therefore, incorporating temporal variations could be crucial to understanding the vertical distributions of soil microbes.

Although bacteria and archaea are abundant in soil, they occupy different ecological niches and are sometimes filtered by different soil variables (Wei et al., 2020). Soil pH was identified as the primary ecological filter for bacterial communities, while soil C/N ratio or salinity were the primary ecological filters for archaeal communities (Auguet, Barberan & Casamayor, 2010; Bates et al., 2011). These differences lead to contrasting vertical distribution patterns between archaea and bacteria, corroborated by previous reports. Bacterial diversity generally decreased towards deeper soil, while archaeal diversity increased along with soil depth in the desert (Wang et al., 2021) and alpine ecosystems (Xu et al., 2021). However, others reported that bacterial and archaeal richness negatively correlated with soil depth in paddy soils (Yuan et al., 2020). The varying conclusions of these studies emphasize the need for further comparisons between the vertical distribution patterns of archaeal and bacterial communities.

Microbial communities typically show a skewed species abundance distribution, with relatively few abundant species co-existing with many rare species (Jousset et al., 2017). Previous studies have indicated that abundant and rare species may possess different functional traits (Jiao & Lu, 2020). Although key roles of abundant microbial taxa in soil function have been well understood, relatively less attention has been paid to rare taxa (Chen et al., 2020). Rare taxa are important reservoirs of genetic diversity, providing functional redundancy and maintaining ecosystem stability (Jousset et al., 2017). In addition, rare taxa may be functionally dissimilar to the abundant members (Liang et al., 2020), offering complementary functions or unique metabolic pathways to support the overall community functioning. Despite their importance, rare species in soil are often neglected due to low sequencing coverages. Therefore, deep sequencing with millions of reads would provide a higher resolution for the detection of rare species and a more comprehensive depiction of the overall soil microbial community along the soil profile.

In the present study, we investigated the vertical and temporal patterns of soil bacterial and archaeal communities in a wheat-soybean rotation agroecosystem at a scale of millions of sequences per sample. We aimed to determine the following: (1) the key determinant from soil depth and temporal fluctuations in shaping bacterial and archaeal communities; (2) differences in the vertical distribution patterns between soil bacteria and archaea; (3) whether rare taxa occupy a larger proportion in the surface soil layer than in the deep soil layer.

## Materials and Methods

### Field description

Our field experiment was carried out at the Chinese Academy of Agricultural Sciences, located in the North China Plain (39.97N, 116.33E), an important food-producing region in China. This region has a warm temperate monsoon climate, with an average annual temperature of 11–13 °C and average annual precipitation of 500–700 mm (80% rainfall occurs from July to August). The soil has a loamy texture and belongs to the fluvo-aquic soil class. The field was fertilized before wheat planting (September 10) under the wheat-soybean rotation system; other agricultural practices, including weeding and tillage, were identical to those done by the local agricultural management.

### Soil sampling and physicochemical analysis

Soil sampling was conducted on October 18 (wheat season) and December 18 (wheat season) of 2019, and on April 17 (wheat season) and August 17 of 2020 (soybean season). In brief, six plots (1 × 1 m) were randomly selected within an area of 20 × 20 m in our study site. For each plot, five soil cores were collected between two plant individuals from a 100 cm long vertical profile that corresponds to depths of 0–20 cm (surface soil layer), 20–50 cm (middle soil layer), and 50–100 cm (deep soil layer). Therefore, each soil sample was a mixture of five soil cores (3.8 cm diameter) for a given soil layer. 72 soil samples (6 plots × 4 sampling times × 3 soil depth) were collected from the six sampling sites. One part of the as-collected soil samples was filtered through a sieve of less than two mm and kept at −80 °C for subsequent extraction of DNA. The other part was sieved with less than one mm and less than 0.25 mm then stored at 4 °C or room temperature to determine the soil physicochemical properties. Soil physicochemical characteristics including soil total carbon (TC), total nitrogen (TN), dissolved organic carbon (DOC), dissolved organic nitrogen (DOC), nitrate (NO${}_{3}^{-}$-N) and ammonium (NH${}_{4}^{+}$-N), moisture, pH, clay, silt, and sand content were determined as described by Bao (2000) and Yang et al. (2017).

### Miseq sequencing and bioinformatics

The soil DNA was extracted using a PowerSoil DNA Isolation Kit (MO BIO Laboratories, USA). The V4 region of the 16s rDNA was amplified using primer pairs 515F/806R (Caporaso et al., 2012). Primer 515F contained a 12 bp barcode unique to each sample for Miseq sequencing detection. All PCR reactions followed Caporaso et al. (2011) within a 25 µL reaction system and were amplified in triplicate. The PCR products were then pooled, purified, and sequenced on an Illumina MiSeq platform at Majorbio Biotech Co., Ltd. (Shanghai, China). The raw sequence data have been deposited on the NCBI SRA (accession No. PRJNA766099).

The raw sequences were trimmed to the shortest sequence length using QIIME 2 Pipeline Version 1.8.0 (Bolyen et al., 2018). Subsequently, the sequences were dereplicated, with all singletons discarded. Sequences were then error-filtered and grouped into amplicon sequence variants (ASVs) using the Deblur software (Amir et al., 2017), with ASVs containing less than two reads removed. Further, potential chimeras were discarded using the Vsearch software. The number of sequences per sample was rarefied to 1,000,000 using the “vegan” package in R. To annotate their taxonomy, the ASVs were blasted against the silva 16s database. Then, the ASV abundance tables were rarefied at 845,591 for bacteria and 10,413 for archaea to ensure even sampling depth within each prokaryote group.

### Data analysis

ASVs with relative abundances below 0.01% were defined as “rare” taxa, while those with relative abundances above 0.1% were defined as “abundant” taxa (Jiao & Lu, 2020). The prokaryotic functional profiles were predicted using the FAPROTAX (Louca, Parfrey & Doebeli, 2016). Soil fertility index is a synthetic variable calculated from the sum of *z*-score transformation of TN, TC, DON, DOC, NH${}_{4}^{+}$-N, and NO${}_{3}^{-}$-N. Bacterial and archaeal alpha-diversity indices, such as richness and Pielou evenness index, were calculated using the “vegan” package (Oksanen et al., 2013). Two-way analyses of variance (ANOVAs) were then performed to examine the effects of soil depth, sampling time, and their interaction on alpha-diversity indices and the proportion of rare ASVs. All data were tested for normality and homogeneity of variance before two-way ANOVAs. A post-hoc test was used to determine paired comparisons among the treatments at a 5% significance. Random forest analysis (Breiman, 2001) was used to explore the soil physicochemical drivers of bacterial and archaeal richness and evenness using the “randomForest” package (Liaw & Wiener, 2002). The “rfPermute” package (Archer, 2016) was then utilized to estimate the significance of important metrics for a random forest model by permuting the response variable.

The effects of soil depth, sampling time, and their interaction on soil bacterial and archaeal community compositions were evaluated using a permutational multivariate analysis of variance (PERMANOVA) with 999 permutations in the “vegan” package (Oksanen et al., 2013). The bacterial and archaeal community compositions were subsequently ordinated using principal coordinates analysis (PCoA) based on the Bray–Curtis dissimilarity matrices in the “vegan” package. A ternary plot was used to demonstrate the distribution of bacterial and archaeal ASVs along the vertical soil profile.

Soil prokaryotic co-occurrence networks for the surface, middle, and deep soil layers were built. Each network was based upon 24 prokaryotic soil communities. ASVs with relative abundances > 0.5% that also occurred in > 50% of the communities were included in the networks to focus solely on abundant ASVs. Spearman’s correlation coefficients were calculated between ASVs using the “Psych” package (Revelle, 2015). *P*-values for multiple tests were calculated using the false discovery rate (FDR), as described by Benjamini & Hochberg (1995). The correlations with a Spearman’s coefficient < 0.8 and a *P*-value > 0.001 were eliminated (Widder et al., 2014). Subsequently, the number of positive and negative correlations, average degree, connectedness, and modularity was calculated in each network using the “igraph” packages. Then, threshold values of Zi and Pi were computed following the reference (Guimera & Amaral, 2005) for each node to determine their topological role. All analyses above were carried out in R (v.3.6.2).

## Results

### Sequencing data analysis and prokaryotic diversity

A total of 185,093,268 reads were obtained after quality control and chimera checks, assigned as 225,608 ASVs. The read numbers were normalized to 1,000,000 for each sample, resulting in a normalized dataset containing 163,862 prokaryotic ASVs. Then, the read numbers of bacteria and archaea were normalized to 845,591 and 10,413 after species annotation, resulting in two normalized datasets containing 162,802 bacterial ASVs and 213 archaeal ASVs. Among these ASVs, 7,382 ASVs (4.5%) occurred in at least half of all samples.

**Figure 1:**
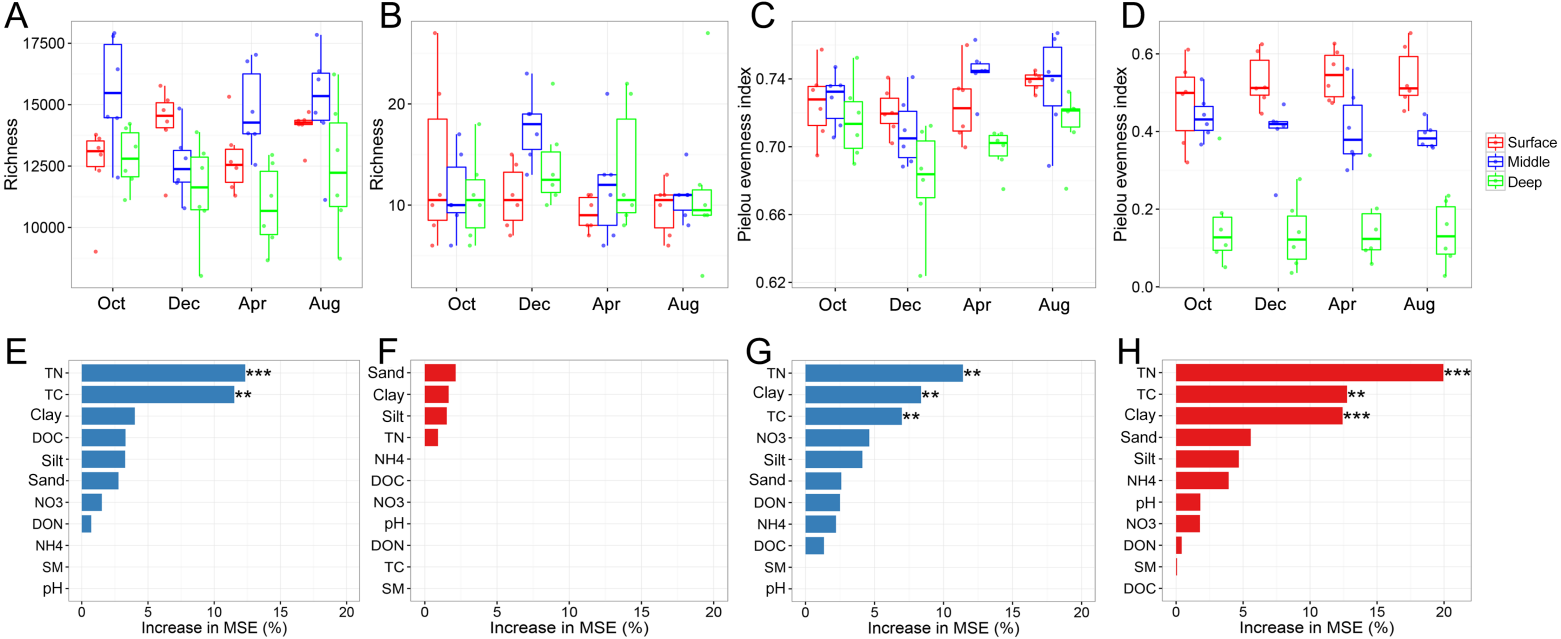
Bacterial and archaeal diversity indices. Bacterial richness (A), archaeal richness (B), bacterial Pielou evenness index (C), and archaeal evenness index (D) among soil layers in October, December, April, and October; random forest mean predictor importance of soil variables on bacterial richness (E), archaeal richness (F), bacterial Pielou evenness index (G), and archaeal Pielou evenness index (H). Significance level: *P* < 0.05, *; *P* < 0.01, **; *P* < 0.01, ***. Abbreviations: TC, total carbon; TN, total nitrogen; DOC, dissolved organic carbon; DON, dissolved organic nitrogen; NO3, nitrate; NH4, ammonium; SM, soil moisture.

The bacterial and archaeal diversities were assessed using the richness and Pielou evenness indices. One of the most interesting findings was that the vertical pattern of soil bacterial and soil archaeal diversity differed. Two-way ANOVA analysis indicated that both bacterial richness and evenness were significantly affected by soil depth (Table 1). Generally, bacterial richness and evenness were lowest in the deep soil layer and peaked in the surface or middle soil layer (Figs. 1A and 1C). In contrast, archaeal richness was unaffected by soil depth, while its evenness showed a decreasing trend along with the soil profile (Figs. 1B and 1D). We did not find an apparent temporal variation of archaeal richness or evenness (Table 1). Although bacterial richness did not show temporal variation (Table 1), its evenness index was significantly higher in August than in December (Fig. 1C). Random forest analysis indicated that TN, TC, and sand content were the key determinants for bacterial and archaeal diversities (except for archaeal richness, Figs. 1E–1H). The soil physicochemical variables are presented in Table S1.

**Table 1:** Two-way ANOVA examining the effects of soil depth and sampling time on bacterial and archaeal richness (S) and Pielou evenness (J) indices.

		Soil depth (D)		Sampling time (T)		D × T	
		*F*	*P*	*F*	*P*	*F*	*P*
Bacteria	S	11.97	<0.001	1.70	0.18	2.11	0.07
	J	13.60	<0.001	5.50	0.002	1.28	0.28
Archaea	S	1.10	0.34	1.60	0.20	1.27	0.28
	J	121.34	<0.001	0.13	0.94	0.63	0.71

### Soil prokaryotic community composition

**Figure 2:**
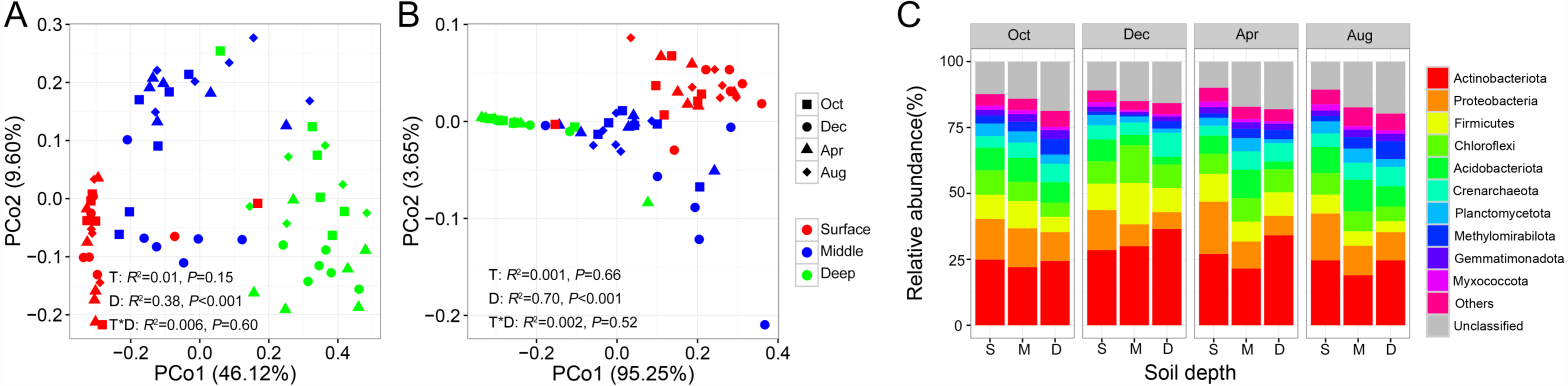
Principal coordinate analysis (PCoA) of soil bacterial community (A) and archaeal community (B); relative abundance of prokaryotic phyla shifts along soil profile (C). Abbreviations: S, surface soil layer; M, middle soil layer; D, deep soil layer.

PERMANOVA analysis indicated that both bacterial and archaeal community compositions were significantly impacted by soil depth (Bacteria: *r*^2^ = 0.38, *P* < 0.001; Archaea: *r*^2^ = 0.70, *P* < 0.001), but unaffected by sampling time and their interaction. This pattern was further evidenced by PCoA ordination based on Bray–Curtis dissimilarity, which indicated that the bacterial and archaeal community compositions were separated by soil depth (Figs. 2A and 2B). We then tested for the temporal variations of the bacterial and archaeal community compositions at each soil depth. We observed that the archaeal soil community did not show any temporal variations at any soil depth (Fig. S1). In contrast, the bacterial soil community composition exhibited strong temporal variation in the middle soil layer (PERMANOVA: *r*^2^ = 0.13, *P* = 0.006, Fig. S1).

At the phylum level, the bacterial community was mainly dominated by Actinobacteria, Proteobacteria, Firmicutes, Chloroflexi, and Acidobacteria, while the archaeal community was mainly dominated by Crenarchaeota in our study site, accounting for 69.19% of the total prokaryotic sequences (Fig. 2C). Next, we investigated the changes in relative abundance patterns of prokaryotic phyla associated with the soil profile. The relative abundance of nearly all phyla was significantly different among soil layers for all sampling times, except for Gemmatimonadetes and Chloroflexi. We observed that Proteobacteria, Firmicutes, Acidobacteria, Planctomycete, and Myxococcota, especially, decreased along with the soil profile, while Actinobacteria and Crenarchaeota exhibited the opposite trend (Fig. 2C, Table S2). Most strikingly, the proportion of unclassified ASVs showed an increasing trend along with the soil profile (Fig. 2C).

**Figure 3:**
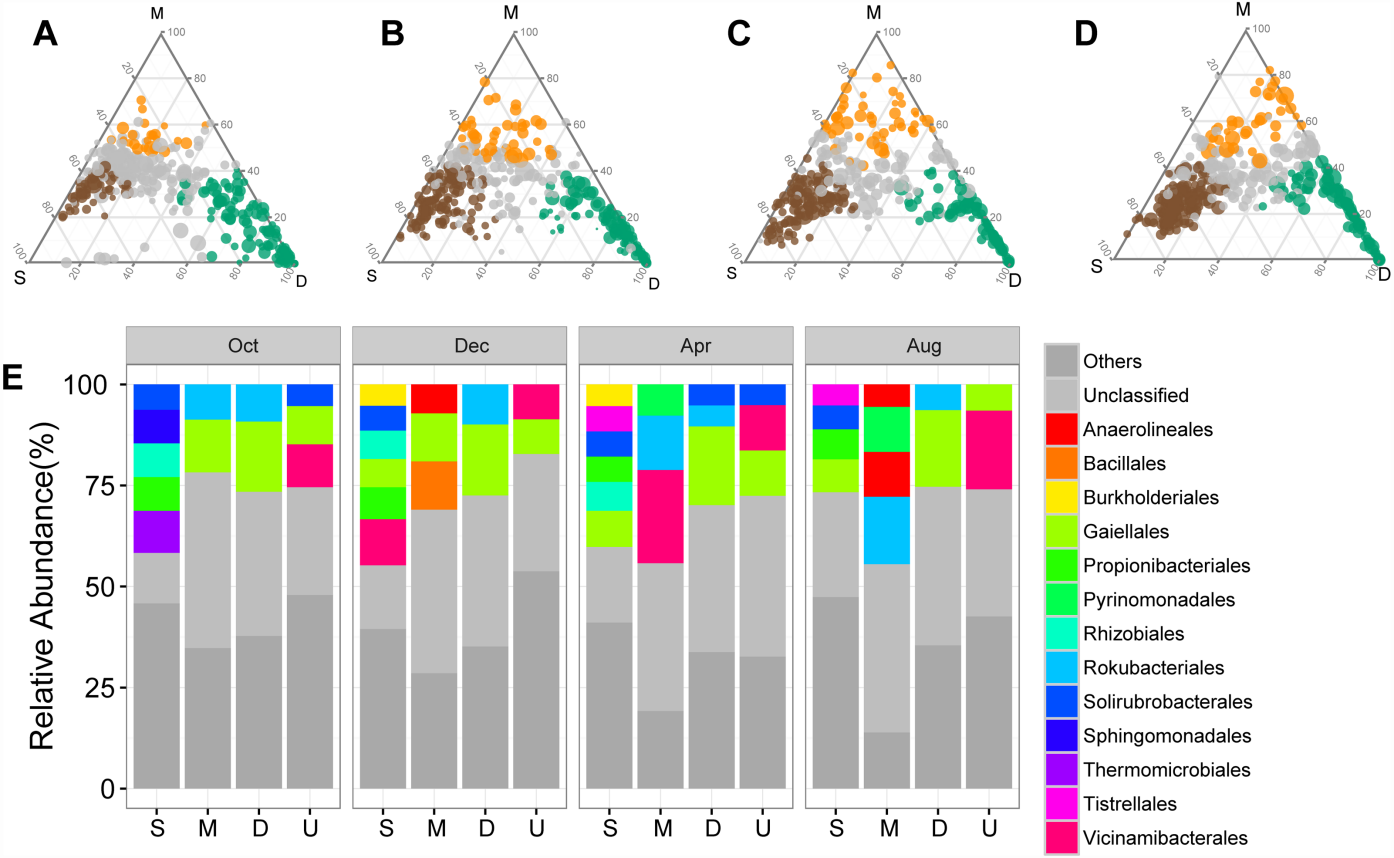
Ternary plots showing the distribution of enriched prokaryotic ASVs in surface (brown), middle (orange), and deep (green) soil layers in October (A), December (B), April (C), and August (D). The size of each circle is equivalent to its relative abundance. The orders of enriched ASVs are displayed in bar plots. Abbreviations: S, ASVs enriched in surface soil layer; M, ASVs enriched in middle soil layer; D, ASVs enriched in deep soil layer; U, unenriched ASVs.

The shifts in bacterial and archaeal communities were also reflected in the abundant ASVs, with 50%–72.49% of total abundant ASVs unevenly distributed along the soil profile among sampling times (Figs. 3A–3D). ASVs enriched in the surface soil layer, mainly Solirubrobacterales and Propionibacterales, accounted for a more significant proportion than those in the middle and deep soil layers (Fig. 3E). ASVs enriched in the middle and deep soil layer, primarily classified as Rokubacterales and Gaiellales, differed from those in the surface soil layer (Fig. 3E). Meanwhile, abundant ASVs also exhibited temporal variations: there were less enriched ASVs (50%) in October than in other sampling times (71.5%–72.5%, Figs. 3A–3D). Interestingly, the proportion of unclassified ASVs was less abundant in the surface soil than in the middle and deep soil layers (Fig. 3E).

### Predicted prokaryotic function

FAPROTAX was adopted to annotate and then screen the key ecological functions of the bacterial communities that contributed to soil biogeochemical cycling. A total of 48 functional groups were identified. Although a minor proportion of all ASVs (5.04%) was assigned to at least one predicted functional group, the predicted functional composition was significantly correlated with the soil prokaryotic community composition as confirmed by the Mantel test (*r* = 0.64, *P* < 0.001). Among these functional groups, we observed that 41 predicted functional groups exhibited significant differences among depths, while 21 functional groups displayed apparent temporal variations (Table S3).

### Rare bacterial and archaeal ASVs

**Figure 4:**
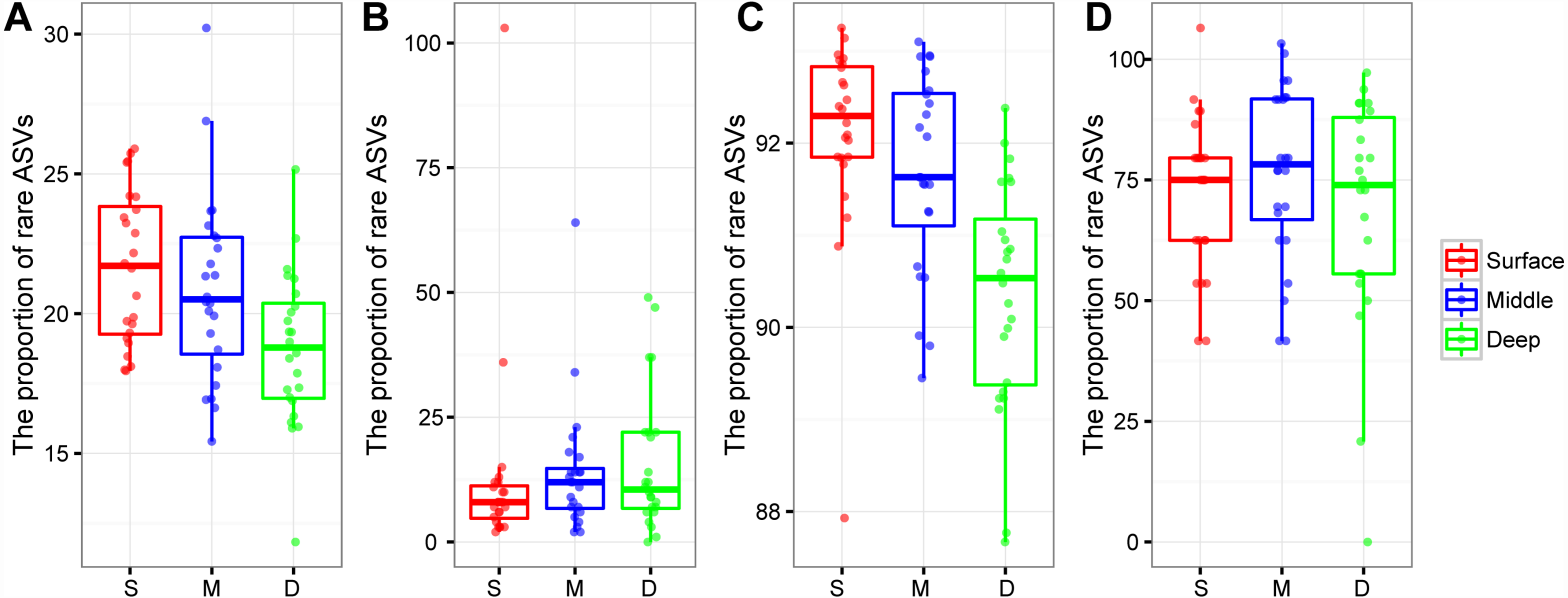
Box plots showing the proportion of rare bacterial richness (A) and rare archaeal richness (B), the proportion of rare bacterial abundance (C), and rare archaeal abundance (D) among soil layers. Abbreviations: S, surface soil layer; M, middle soil layer; D, deep soil layer.

The proportion of rare bacterial richness and abundance was highest in the surface soil layer and showed a decreasing trend along with the soil profile (Figs. 4A and 4C). On the other hand, the proportion of rare archaeal richness and abundance were not impacted by soil depth (Figs. 4B and 4D). Random forest analysis further indicated that the rare bacterial community was the key determinant of the soil fertility index and functional diversity (Fig. S2). Moreover, the potential role of 43 archaeal and bacterial phyla on soil fertility was assessed. Among these phyla, Latescibacterota, Zixibacteria, Dadabacteria, Dependentiae, Firmicutes, Micrarchaeota, and Calditrichota were the main predictors of the soil fertility index (Fig. S3); these phyla, except Firmicutes, were relatively rare taxa, accounting for 0.00034%–0.31% of the total reads. We further explored the potential functions of rare phyla through FAPROTAX: Desulfobacterota accounted for 90.65% of sulfate respiration and 46.26% of iron respiration; Cyanobacteria accounted for 6.10% of nitrogen fixation and 97.50% of phototrophy; Halanaerobiaeota accounted for 62.90% of cellulolysis; and Euryarchaeota and Halobacterota accounted for 14.33% and 0.12% of dark hydrogen oxidation, respectively.

### Prokaryotic co-occurrence network

**Figure 5:**
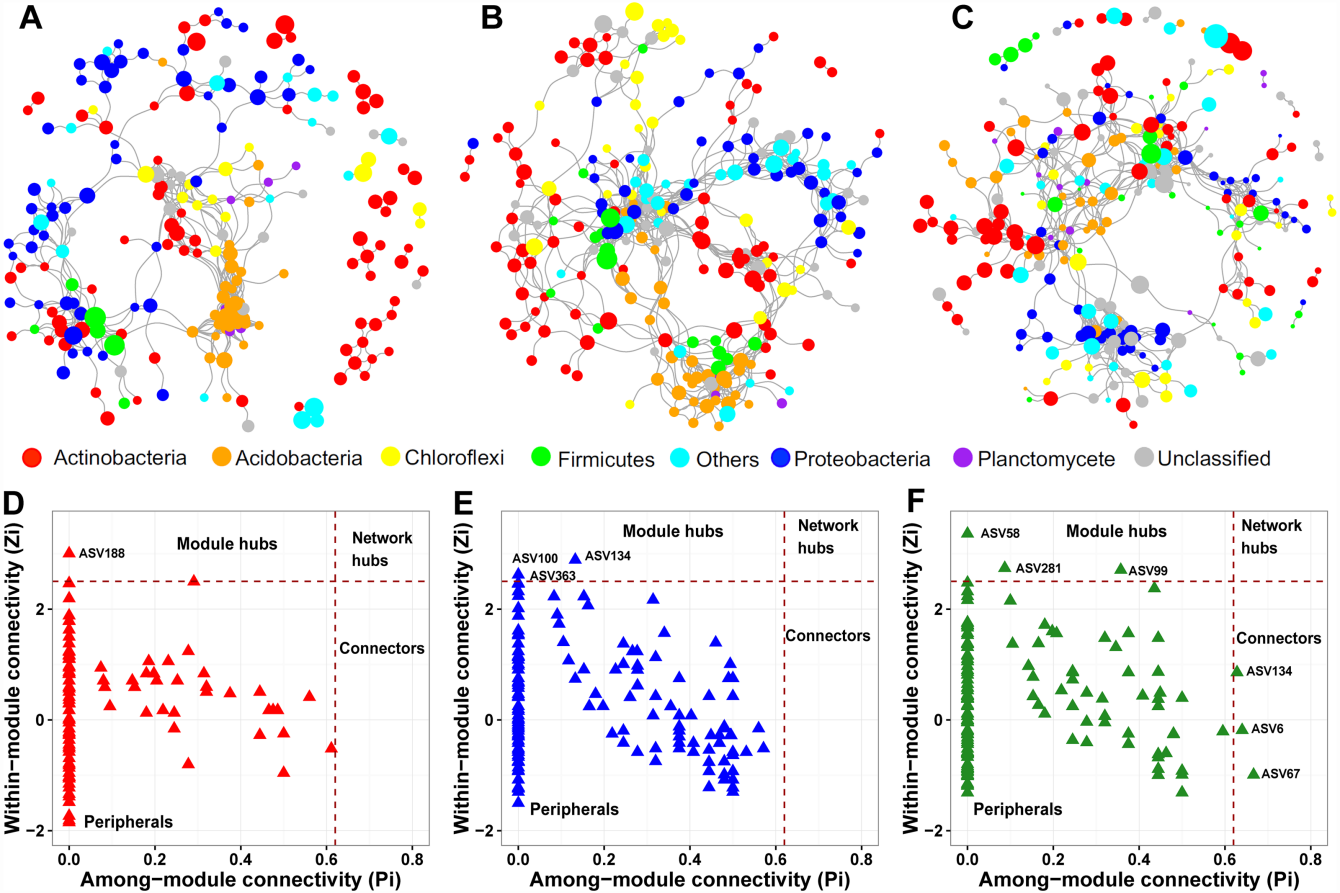
Prokaryotic co-occurrence networks in surface (A), middle (B), and deep (C) soil layers. The size of each node is proportional to its relative abundance. Pi-Zi plots showing the distribution of prokaryotic soil ASVs based on their topological roles in surface (D), middle (E), and deep (F) soil layers.

We constructed a soil prokaryotic co-occurrence network for each soil layer. The network size generally became larger along with increasing soil depth, with 214, 247, and 274 nodes in the surface, middle, and deep soil layers, respectively (Figs. 5A–5C). Additionally, we observed that only a small proportion of nodes (54) were shared among these three networks. The network of the surface soil layer was analogous to the network of the middle soil layer (153 nodes shared) but was quite dissimilar to that of the deep soil layer (65 nodes shared). The surface and middle soil layers’ networks were more connected than that of the deep soil layer (Figs. 5A–5C). This pattern was affirmed by their topological characteristics (Table S4). Furthermore, the number of positive/negative links gradually decreased along with the soil profile (Table S4).

From the plot of Zi (a value measuring within-module connectivity) against Pi (a value measuring among-module connectivity), the different roles of each node in the network were identified (Figs. 5D–5F). Notably, there were more keystone species observed in the deep soil layer (six) than in the surface (one) and middle (three) soil layers (Figs. 5D–5F). These keystone species were not shared among the three networks. The classification of these keystone species is listed in Table S5.

## Discussion

### Different vertical patterns between bacterial and archaeal diversities

We observed that the deep soil layer harbors the lowest bacterial diversity, which is consistent with previous studies from the alpine meadow (Xu et al., 2021), soybean cropland (Hao et al., 2020), and deciduous birch forest (Mundra et al., 2021). This finding confirmed the ecological principle that more extreme environments are expected to be inhabited by a less diverse community of microbes (Gaston, 2000). In contrast to previous studies, we observed that bacterial richness and evenness in the middle soil layer were roughly equal to that in the surface. This observation may have the following two explanations. First, this is likely attributable to the soil carbon content which did not differ between the surface and middle soil layer. Soil organic carbon content is the most important factor that influences soil microorganisms in addition to pH (Fierer, 2017; Kukreti et al., 2020) and was observed to be the best predictor of bacterial diversity, as indicated by random forest analysis in this study. Second, on a depth-weighted basis, the microbial biomass in the middle soil layer may not be less than that in the surface soil layer, thus, containing an equal diversity to the surface soil layer.

For archaea, its richness was unaffected by soil depth and cannot be explained by any measured soil variable. Archaea are reported to be highly adaptable to environmental stress (Cao et al., 2012), so may survive in the nutrient-poor and oxygen-deficient deep soil layer. In addition, we detected that archaeal communities are highly uneven in deep soil, meaning that there is extreme dominance by one or a few archaeal taxa. Therefore, the archaeal communities in deep soil would be less resistant to environmental stress (Wittebolle et al., 2009).

### Vertical and temporal patterns of bacterial and archaeal communities

Both bacterial and archaeal community compositions exhibited distinct distribution patterns with the vertical soil profile, mainly due to the drastic differences in soil carbon and nitrogen content and soil texture (Jobbágy & Jackson, 2000; Eilers et al., 2012; Gu et al., 2017). We also detected different ecological preferences for certain phyla. For instance, Actinobacteria and Crenarchaeota showed increasing abundance along with the vertical soil profile. This result corresponds with the conclusions of previous studies that archaea occupy a larger proportion in the deep soil layer due to their anaerobic characteristic. However, some bacterial phyla, including Proteobacteria, Firmicutes, and Acidobacteria, displayed a sharply decreasing trend in the deep soil layer. This trend may be owing to the copiotrophic-oligotrophic trade-off theory (Ramirez, Craine & Fierer, 2012) and/or the deficiency in oxygen availability. For instance, Proteobacteria are typically copiotrophic and capable of degrading various organic materials (Eilers et al., 2012). Thus, the higher abundances of Proteobacteria in the surface layer may, in part, reflect increased organic carbon availability.

We only detected a slight temporal variation in bacterial and archaeal community compositions. In a meta-analysis, Shade et al. (2013) found that soil microbial communities were consistently less temporally variable than other ecosystems. For time-series field studies, it is impossible to collect samples in the exact same location due to destructive soil sampling. Therefore, soil microbial community dynamics may be masked by the high small-scale spatial heterogeneity (Docherty et al., 2015). Although the use of the soybean in a crop rotation would increase soil total nitrogen, there is no apparent difference in soil TN and TC among seasons (Table S6) in the present study, perhaps due to the removal of crop residues after harvest. As proposed by previous studies, temporal variations of soil microbial communities are driven by soil physicochemical characteristics (Yang et al., 2017). Consequently, the minimal temporal variations might be explained, in part, by the unapparent difference in soil TC and TN. Alternatively, soil contains many dormant microbes (Lennon & Jones, 2011), which were indiscriminately captured using the 16s rRNA sequencing approach (Shade et al., 2013). For the reasons above, the soil prokaryotic communities only fluctuated within a narrow range over the given time period.

### Vertical pattern of rare ASVs and their potential function

Deep sequencing enabled us to explore reliable rare taxa in soil. For the first time, the vertical patterns of rare bacterial and archaeal taxa were evaluated. We observed that the proportion of rare bacterial ASVs decreased along with the soil profile. The root system in the surface soil layer was more abundant than that in the middle or deep soil layers (Chaparro, Badri & Vivanco, 2014) and may provide more niches for rare species with specialized functions. Alternatively, it was proposed that rare species may occur from stochastic processes (Jousset et al., 2017), whereas deterministic processes would reduce rare species. Since soil nutrient availability generally decreased along with the vertical soil profile, stochastic processes would play a less predominant role in the deep soil layer.

Microbial rare taxa constitute an important “genomic reservoir” and could be used for a variety of functions (Jousset et al., 2017). It was evidenced that rare taxa contributed to sulfate reduction (Pester et al., 2012) and have the potential for bioremediation (Pascoal, Magalhães & Costa, 2020). Recently, the ecological importance of rare taxa in terrestrial and aquatic ecosystems has been increasingly recognized (Jousset et al., 2017; Chen et al., 2020). In the present study, the community of rare taxa was observed to be a key determinant of soil fertility and ecosystem multifunctionality, indicating that rare taxa contribute greatly to agroecosystem functioning. Likewise, Chen et al. (2020) also observed that rare bacterial and fungal taxa (less than 3% of total reads) were the major drivers of ecosystem multifunctionality in long-term fertilized soils. As revealed by Random Forest analysis, rare prokaryotic phyla (*e.g.*, Latescibacterota, Zixibacteria, and Dadabacteria) had an over-proportional role in the soil biogeochemical cycling process. In contrast, the dominant bacterial and archaeal phyla (*e.g.*, Proteobacteria, Actinobacteria, and Acidobacteria) showed little control over soil fertility. Our analysis further revealed that rare phyla influence narrow-range functions, such as dark hydrogen oxidation, sulfate respiration, and cellulolysis. Other studies highlighted that rare taxa exert great influence over sulfate reduction (Pester et al., 2010; Dell’Anno et al., 2012) and organic pollutant degradations, where their removal greatly impacted ecosystem function (Jousset et al., 2017). Taken together, our knowledge of the ecological role of the rare microbial taxa is still obscure with more attention needed.

### Vertical patterns of prokaryotic co-occurrence networks

Our results emphasized that the prokaryotic co-occurrence network was more complex in the surface and middle soil layers than in the deep soil layer, reflected by the higher connectedness and average degree. Likewise, Xu et al. (2021) observed a more complex and more extensive microbial network in the surface than in the deep soil layer of meadows and shrubland. Higher network complexity in the surface and middle soil layer might be explained, in part, by greater amounts of substrate and nutrient in the surface soil layer (Creamer et al., 2016), as microbial interactions feeding on these substrates would be largely strengthened. In fact, a large number of studies have found that soil microbial networks would be more connected after nutrient amendments (Yang et al., 2019; Yang et al., 2020). Moreover, the microbial richness and biomass were extremely low in the deep soil layer (Eilers et al., 2012; Xu et al., 2021), reducing the opportunity for different species to interact. It has been proposed that complex networks contribute to better environmental adaption and higher efficiency of resources transfer than simple networks (Morriën et al., 2017). In this sense, the complex prokaryotic networks in the surface and middle soil layers would lead to better biogeochemical functions and provide a stable soil environment for plant growth.

We observed a gradual increase in proportion of negative links along the soil profile by assessing the correlations among microbial species, which indicates that microbial competition for the same resources increases with soil depth. The niche differentiation would be a possible explanation for this result. The surface and middle soil layers are easily impacted by external disturbance; the roots within these layers also create heterogeneous environments (Morriën et al., 2017), leading to significant niche differentiation and reduction of competition. In contrast, the deep soil layer is deficient in roots and is less impacted by external disturbance, possessing a more homogeneous environment and weak niche differentiation (Faust & Raes, 2012). The nutrient-limited environment in the deep soil layer would also induce intense competition among species.

In conclusion, we fully evaluated the vertical and temporal patterns of soil bacterial and archaeal communities and co-occurrence networks in a wheat-soybean rotation agroecosystem. Our results showed that soil depth as a more predominant factor than sampling time in shaping soil prokaryotic communities and co-occurrence networks and revealed different vertical distribution patterns of bacteria and archaea. With deep sequencing at a depth of millions of sequences per sample, we can investigate the rare microbial taxa and reveal their potential in soil biogeochemical function. Overall, our better understanding of vertical and temporal patterns of soil prokaryotic communities should enable more strategic agricultural management to mitigate adverse environmental impacts and improve crop productivity.

## Supplemental Information

10.7717/peerj.12868/supp-1 Table S1 Soil physiochemical variables among soil layers in October, December, April and August

10.7717/peerj.12868/supp-2 Table S2 Pearson correlations between soil variables and main soil prokaryotic phyla

10.7717/peerj.12868/supp-3 Table S3 Two-way ANOVA examining the effects of soil depth and sampling time on predicted ecological function of prokaryotic communities

10.7717/peerj.12868/supp-4 Table S4 Co-occurrence network topological parameters Major topological properties of the soil prokaryotic networks in surface, middle and deep soil layers.

10.7717/peerj.12868/supp-5 Table S5 The classification at each taxonomic level of the keystone species

10.7717/peerj.12868/supp-6 Table S6 Two-way ANOVA examining the effects of soil depth and sampling time on soil moisture (SM), pH, total nitrogen (TN), total carbon (TC), ammonia (NH4+-N), nitrate (NO3–N), dissoved organic carbon, dissoved organic nitrogen, sand, silt and clay content

10.7717/peerj.12868/supp-7 Figure S1 Supplemental Figures

## Additional Information and Declarations

### Data Availability

The following information was supplied regarding data availability: The raw sequence data is available at NCBI SRA: PRJNA766099.
