# Supplementary material for: Vertical and temporal variations of soil bacterial and archaeal communities in wheat-soybean rotation agroecosystem
Source: PeerJ. 2022 Feb 10;10:e12868. doi: 10.7717/peerj.12868 (PMC8841036; doi:10.7717/peerj.12868)
Supplement: Figure S1 [file peerj-10-12868-s007.docx]

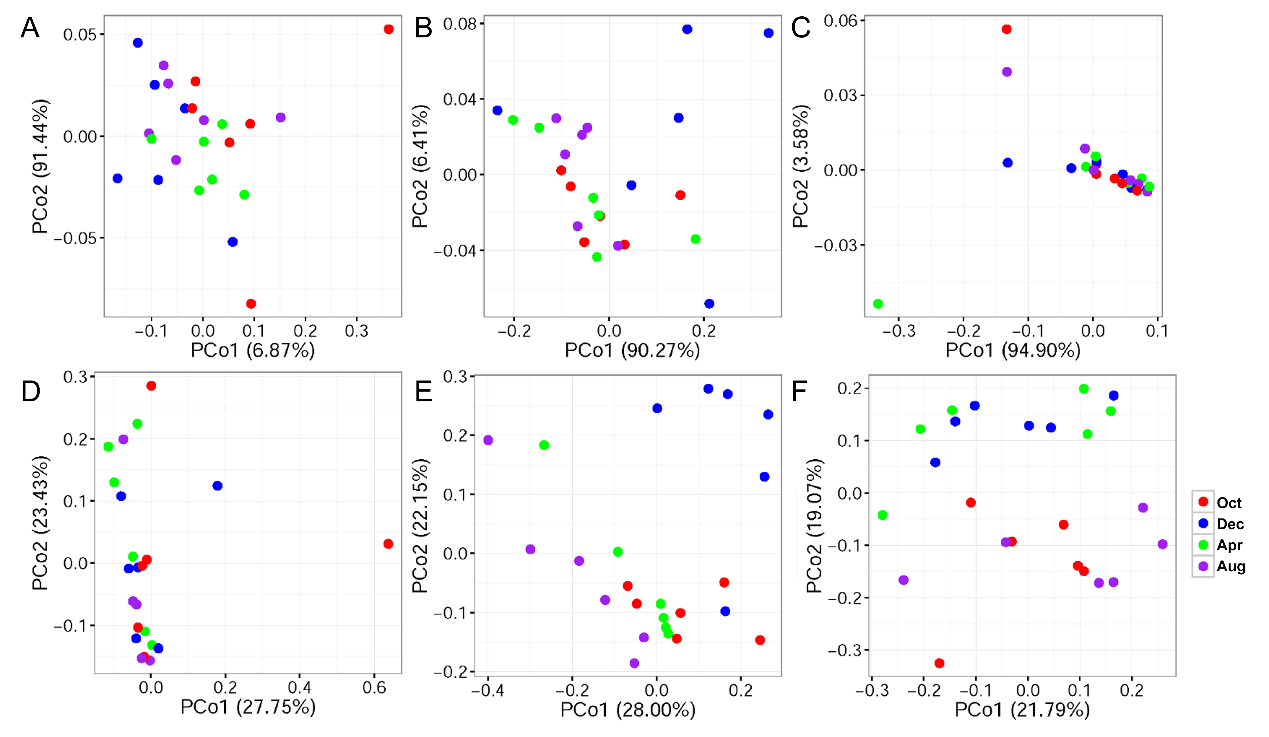


**Fig. S1.** Principal coordinate analysis (PCoA) of soil archaeal community in surface (A), middle (B) and deep (C) soil layer among seasons; principal coordinate analysis (PCoA) of soil bacterial community in surface (D), middle (E) and deep (F) soil layer among seasons.


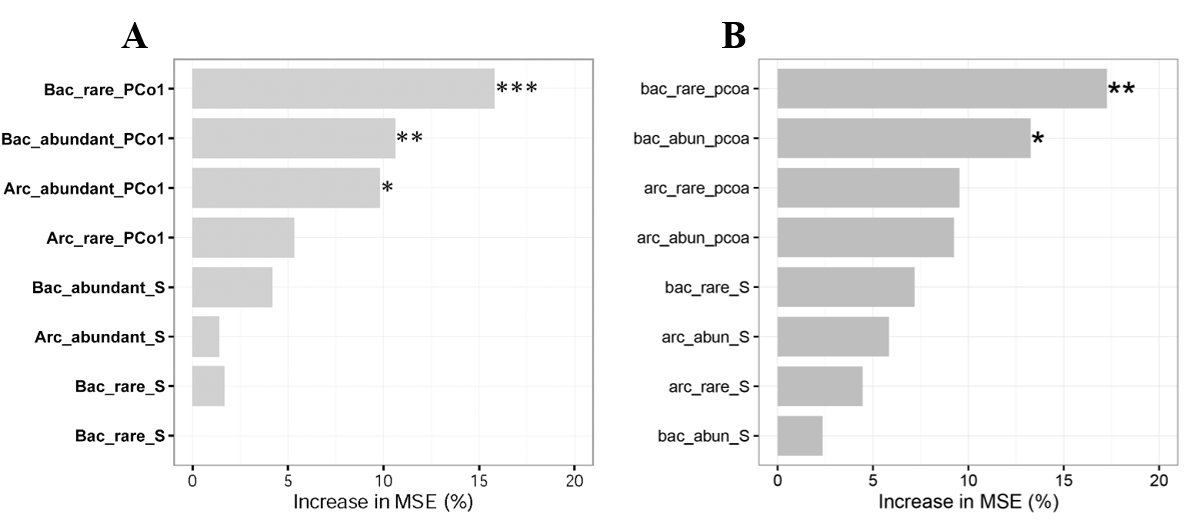


**Fig. S2.** Random forest mean predictor importance of PCo1 of rare bacterial community (Bac_rare_PCo1), PCo1 of abundant bacterial community (Bac_abundant_PCo1), PCo1 of rare archaeal community (Arc_rare_PCo1), PCo1 of abundant archaeal community (Arc_abundant_PCo1), rare bacterial richness (Bac_rare_S), abundant bacterial richness (Bac_abundant_S), rare archaeal richness (Arc_rare_S), abundant archaeal richness (Arc_abundant_S) on soil fertility index (A) and functional diversity (B). Significance level: *P* < 0.05, *; *P* < 0.01, **; *P* < 0.01, ***.


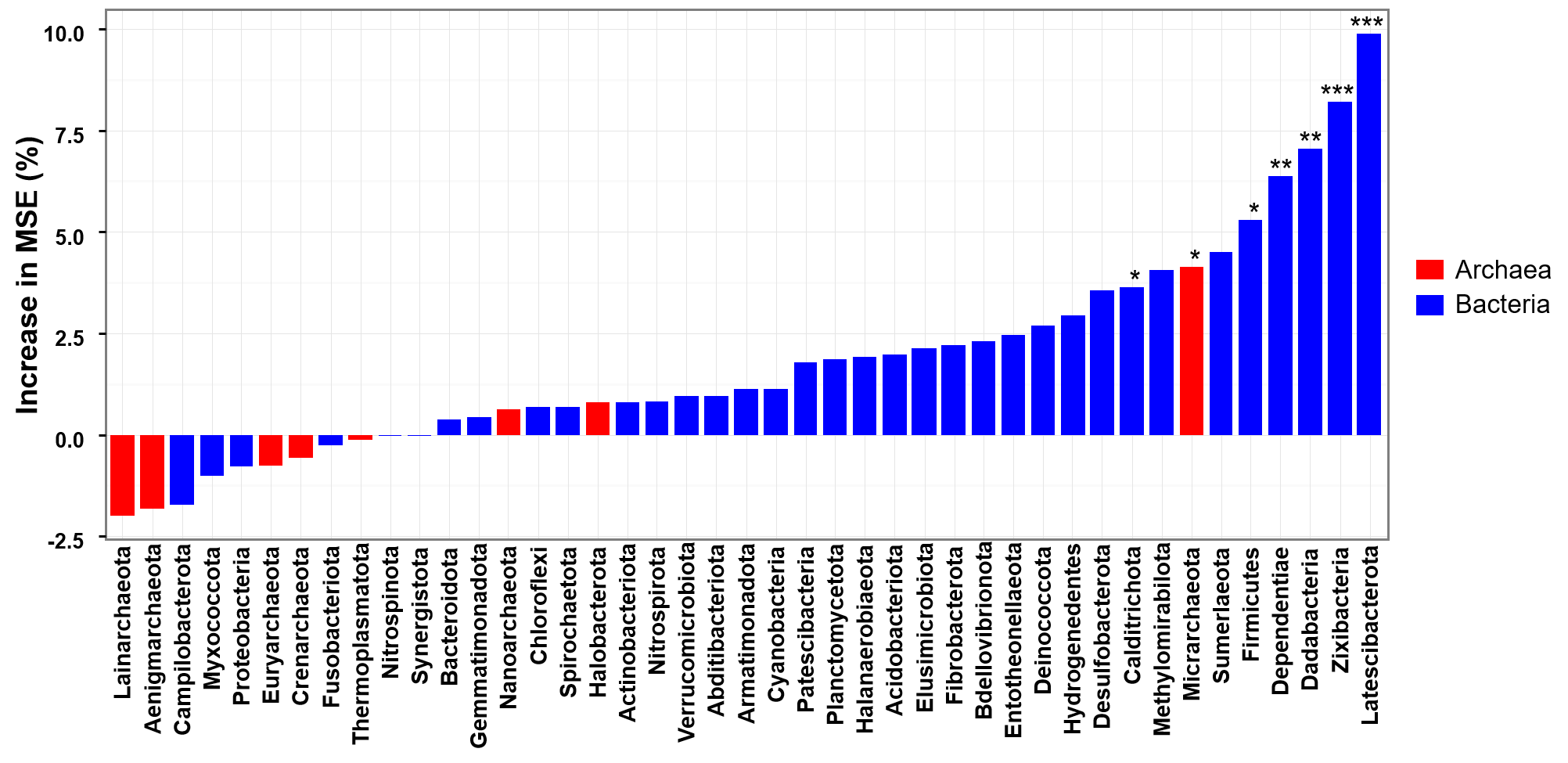


**Fig. S3.** Random forest mean predictor importance of prokaryotic phyla on soil fertility index. Significance level: *P* < 0.05, *; *P* < 0.01, **; *P* < 0.01, ***. Abbreviations: TC, total carbon; TN, total nitrogen; DOC, dissolved organic carbon; DON, dissolved organic nitrogen; NO3, nitrate; NH4, ammonium; SM, soil moisture.
